# In vitro Cariostatic effects of cinnamon water extract on nicotine-induced *Streptococcus mutans* biofilm

**DOI:** 10.1186/s12906-020-2840-x

**Published:** 2020-02-11

**Authors:** Abdulaziz M. Alshahrani, Richard L. Gregory

**Affiliations:** 1 Department of Restorative Dental Sciences, College of Dentistry, King Khalid University, Abha, Saudi Arabia; 2 Department of Cariology, Operative Dentistry and Dental Public Health – Cariology and operative dentistry, Indiana University School of Dentistry, Indianapolis, IN 46202 USA; 3 Department of Biomedical Sciences and Comprehensive Care, Indiana University School of Dentistry, Indianapolis, IN 46202 USA

**Keywords:** Oral Bacteria, Streptococcus mutans, Caries, Cinnamon, Plaques, Biofilms. Tobacco

## Abstract

**Background:**

Dental caries is one of the most prevalent chronic oral diseases worldwide. Dental caries is mainly associated with *Streptococcus mutans* and the *Lactobacillus* species. A specific relationship was found between nicotine and *S. mutans* growth as the presence of nicotine increased *S. mutans* biofilm formation. Nicotine is able to increase the number of *S. mutans* and extracellular polysaccharide (EPS) synthesis. Among the widely used herbs and spices is cinnamon which demonstrated a strong antibacterial activity against a wide variety of bacteria including *S. mutans* and showed the ability to inhibit *S. mutans* biofilm formation. Cinnamon essential oil, obtained from the leaves of *C. zeylanicum*, has been demonstrated to be effective against *S. mutans* and *Lactobacillus acidophilus*, which are partially responsible for dental plaque formation and caries development. The aim of this study was to identify the effects of nicotine exposure on the inhibitory effects of cinnamon water extract on *S. mutans* biofilm formation.

**Materials and methods:**

A 24-h culture of *S. mutans* UA159 in microtiter plates was treated with varying nicotine concentrations (0–32 mg/ml) in Tryptic Soy broth supplemented with 1% sucrose (TSBS) with or without a standardized concentration (2.5 mg/ml) of cinnamon water extract. A spectrophotometer was used to determine total growth absorbance and planktonic growth. The microtiter plate wells were washed, fixed and stained with crystal violet dye and the absorbance measured to determine biofilm formation.

**Results:**

The presence of 2.5 mg/ml cinnamon water extract inhibits nicotine-induced *S. mutans* biofilm formation from 34 to 98% at different concentrations of nicotine (0–32 mg/ml).

**Conclusion:**

The results demonstrated nicotine-induced *S. mutans* biofilm formation is decreased from 34 to 98% in the presence of 2.5 mg/ml cinnamon water extract. This provides further evidence about the biofilm inhibitory properties of cinnamon water extract and reconfirms the harmful effects of nicotine.

## Background

Dental caries is considered one of the most widespread chronic bacterial infections in the world [1]. *Streptococcus mutans* and *Lactobacilli* are considered the main bacteria involved in dental caries. However, other bacteria are involved in the caries process such as Actinomycetes and *Veillonella species.* The etiology of dental caries is attributed to differences in eating habits, especially sugar consumption, oral hygiene practices, the virulence of oral bacteria, and alterations in oral protective mechanisms. According to National Health and Nutrition Examination Survey (NHANES) 2015–2016, the prevalence of total and untreated dental caries in primary or permanent teeth among youth aged 2–19 years was 45.8 and 13.0%, respectively [2]. In 2007 approximately 91% of adults in the US older than 20 years had dental caries and 27% had untreated carious lesions [3].

Nicotine is the most abundant alkaloid present in Tobacco. Moreover, Tobacco addiction is primarily caused by nicotine as it is considered the main biobehavioral chemical compound found in tobacco that explains the reason for habitual tobacco use [4]. The relation between smoking and dental caries has been extensively investigated. A positive correlation has also been found between nicotine and *S. mutans* biofilm. Nicotine upregulates certain *S. mutans* virulence genes including GTF and glucan binding protein (GbpA) expression, and Ldh, nlmC, and phosphotransferase system (PTS)-associated genes, which eventually leads to more lactic acid production [5, 6]. A recent in-vivo study indicates that caries was higher in Wistar rats infected with *S. mutans* and treated with nicotine compared with a nicotine-untreated group [7].

Dental caries is a dynamic process of demineralization and remineralization cycles that affects the quantity of minerals in the tooth structure. Many approaches have been used to arrest or reverse the demineralization process such as fluoride, casein phosphopeptide (CPP) amorphous calcium-phosphate (ACP) complexes and Silver diamine fluoride which have shown the ability to inhibit demineralization and to enhance the remineralization process [8–10].

Recent focus has been on decreasing the incidence of caries by reducing the load of *S. mutans* and dental plaque. Various antibacterial compounds have been used for these purposes, such as chlorhexidine, xylitol, triclosan, cetylpyridinium chloride, sanquinarin, sodium dodecyl sulphate, and various metal ions (tin, zinc, copper) [11]. With the exception of chlorhexidine, the effectiveness of these agents is still controversial [12]. Chlorhexidine has been widely prescribed; however, several side effects have been reported for this agent [13]. For that reason, research has been conducted to seek adjunctive antibacterial compounds that could prevent or reduce plaque formation on tooth surfaces.

Among the widely used herbs and spices is cinnamon from *Cinnamomum* species, which has shown antimicrobial activity against pathogens and assists in the preservation of food [14]. Cinnamon demonstrated a strong antibacterial activity against a wide variety of bacteria including *Streptococcus faecalis* DC 74, *Pseudomonas aeruginosa* ATCC 27859, *Enterobacter cloacae* ATCC 13047, *Staphylococcus aureus* 6538 P and *Enterococcus faecalis*, one of the main causative factors of pulp and periapical diseases of the oral cavity [15, 16] .Cinnamon essential oil, obtained from the leaves of *Cinnamomum zeylanicum*, has been shown to be effective against *S. mutans* and *Lactobacillus acidophilus*, which are partially responsible for dental plaque formation and caries development [17]. A number of studies have investigated the effect of cinnamon water extract on *S. mutans* and demonstrated that cinnamon water extract was able to inhibit biofilm formation [18]. However, no study has investigated the effect of cinnamon water extract specifically on nicotine-induced *S. mutans*.

Cinnamon is a very commonly used specie in the middle east and southeast Asian cultures where it is added to savory dishes, as well as to beverages in which cinnamon powder is mixed with water which is basically a water extract of cinnamon. In some of those cultures, people enjoy drinking cinnamon while smoking cigarettes, so if cinnamon water extract is proven to be a cariostatic agent it could be incorporated into oral hygiene products with the appropriate anti-*S. mutans* biofilm concentration which have no cytotoxicity, so both smokers and non-smokers may gain a dual benefit of a mouth-refreshing and a caries-preventing agent. Currently, cinnamon is added to oral hygiene products just as a flavoring agent and mouth refresher.

This study utilized *S. mutans* as it is the most common bacterium associated with the initiation of dental caries and most retentive in different oral cavity sites whereas lactobacilli flourish in a carious environment and contribute to caries progression. Lactobacilli are less retentive in oral cavity sites and present in high numbers as free-floating planktonic bacteria [19]. For these reasons, *S. mutans* was selected to be tested in this study. This study aims to investigate the cariostatic effects of Cinnamon water extract and effects of nicotine exposure on the inhibitory effects of cinnamon water extract on *S. mutans* biofilm formation.

## Methods

### Determination of MIC and MBIC of cinnamon water extract

Before starting the main study, a preliminary experiment was conducted to determine the minimum inhibitory concentration (MIC) and the minimum biofilm inhibitory concentration (MBIC) of cinnamon water extract alone on the growth of *S. mutans* in tryptic soy broth supplemented with 1% sucrose (TSBS). A recent pilot study report indicated that the MIC and MBIC of cinnamon water extract was 5 mg/ml [18]. A 16 h culture of *S. mutans* UA159 (ATCC 700610) was grown in TSB at 37 °C in 5% CO_2_ and stored with 20% glycerol at -80 °C.

### Chemicals and reagents

The preliminary experiment was accomplished by mixing 20 mg/ml of 100% pure *Cinnamomum burmannii* powder, also known as Indonesian cinnamon, or korintje (Morton & Bassett Spices, Rohnert Park, CA) in sterile deionized water, the solution was transferred into a test tube and heated for 60 min at 121 °C at 15 PSI of pressure in an autoclave to sterilize the cinnamon solution. The sterility of the solution was confirmed by streaking on a blood agar plate.

### Extract preparation

After the sterility of the solution was confirmed the solution (cinnamon powder mixed with sterile deionized water) was centrifuged (Beckman GS-6R Refrigerated Centrifuge) for 10 min to clarify the preparation. Then, dilutions of cinnamon water extract in the supernatant were prepared.

### Preparation of the different concentrations and MIC and MBIC determination

Serial dilutions of cinnamon water extract in the supernatant (ranging in concentration from 0 to 10 mg/ml) were prepared in TSBS. Ten μl of a 16 h culture of *S. mutans* (approximately 10^6^ colony-forming units [CFU]/ml, determined by spiral plating) in TSB was treated with 190 μl of the cinnamon water extract dilutions and incubated at 37 °C for 24 h in sterile 96-well flat-bottom microtiter plates (Fisher Scientific, Newark, DE, USA). The optical density (OD) values of the bacterial cultures were measured at 595 nm in a spectrophotometer (SpectraMax 190; Molecular Devices, Sunnyvale, CA, USA). The MIC was determined by the concentration where there was an obvious clear-cut decrease in the absorbance. After incubation, the unbound planktonic cells (120 μl) were gently aspirated and transferred to a new 96-well plate and the OD at 595 nm was determined in order to calculate the effect on planktonic cells. The remaining planktonic cells were removed from the biofilm microtiter plate wells (leaving attached biofilm), and 200 μl of 10% formaldehyde was added to each well for 30 min to fix the cells. After 30 min, the formaldehyde was removed, and the biofilm cells were washed 3 times with deionized water. Two hundred μl of 0.5% crystal violet dye was added to each well and the cells stained for 30 min. The wells were rinsed three times and 200 μl of 2-isopropanol was placed into each well for 1 h to lyse the cells and extract the crystal violet. The plates were read in a spectrophotometer at 490 nm to measure biofilm formation [20].

### Inhibition of nicotine-induced biofilm formation by cinnamon water extract

The MIC for cinnamon water extract was determined to be 2.5 mg/ml. A stock solution of cinnamon water extract at 2.5 mg/ml was prepared in the same manner in the preliminary experiments. In order to measure the effect of cinnamon water extract on nicotine-treated *S. mutans* initial biofilm formation, serial dilutions of TSBS were prepared to yield 0, 0.25, 0.5, 1, 2, 4, 8, 16 and 32 mg/ml nicotine (Sigma-Aldrich Chemical Co., St. Louis, MO) without cinnamon water extract, and 0, 0.25, 0.5, 1, 2, 4, 8, 16 and 32 mg/ml nicotine with the MIC dilution of cinnamon water extract (2.5 mg/ml; determined by the results of the preliminary experiment). One hundred ninety μl of TSBS at each nicotine concentration was aliquoted into wells of a sterile 96-well flat bottom microtiter plate. Ten μl of a fresh overnight TSB culture of *S. mutans* was added to each well. The microtiter plate was incubated in 5% CO_2_ at 37 °C for 24 h. The following day, the total absorbance (biofilm and planktonic growth) was measured in a spectrophotometer (SpectraMax 190; Molecular Devices Inc., Sunnyvale, CA) at 595 nm. Next, 120 μl from each well was transferred to the corresponding well of a new microtiter plate. The absorbance of each well was read at 595 nm to measure planktonic growth. The remaining planktonic cells were removed from the biofilm microtiter plate wells (leaving attached biofilm), and 200 μl of 10% formaldehyde was added to each well for 30 min to fix the cells. After 30 min, the formaldehyde was removed, and the biofilm cells were washed 3 times with deionized water. Two hundred μl of 0.5% crystal violet dye was added to each well and the cells were stained for 30 min. The wells were rinsed 3 times and 200 μl of 2-isopropanol was placed into each well for 1 h to lyse the cells and extract the crystal violet. The plates were read in a spectrophotometer at 490 nm to measure biofilm formation. Controls included biofilms of *S. mutans* without nicotine and with or without 2.5 mg/ml cinnamon water extract as well as a sterility control.

### Statistical analysis

Each experiment was repeated three times (Additional file 1). A two-way ANOVA was used to compare the effects of cinnamon water extract exposure (each dilution individually), on nicotine concentration (0, 0.25, 0.5, 1.0, 2.0, 4.0, 8.0, 16.0 and 32.0 mg/ml) and their interaction on planktonic, biofilm, and total growth. Pairwise comparisons were achieved using Fisher’s Protected Least Significant Differences method to control the overall significance level at 5% in which comparisons were made between nicotine concentrations to the zero nicotine both with and without cinnamon water extract. Pairwise comparisons were also made comparing each nicotine concentration with and without cinnamon water extract. The comparisons involving cinnamon water extract exposure were of primary interest to test the study hypotheses. The distribution of the measurements was investigated and found to be non-normal, and thus a rank transformation was used to satisfy the ANOVA assumptions prior to analysis.

## Results

### Results of the preliminary experiment; effect of cinnamon water extract on S. mutans

**Fig. 1:**
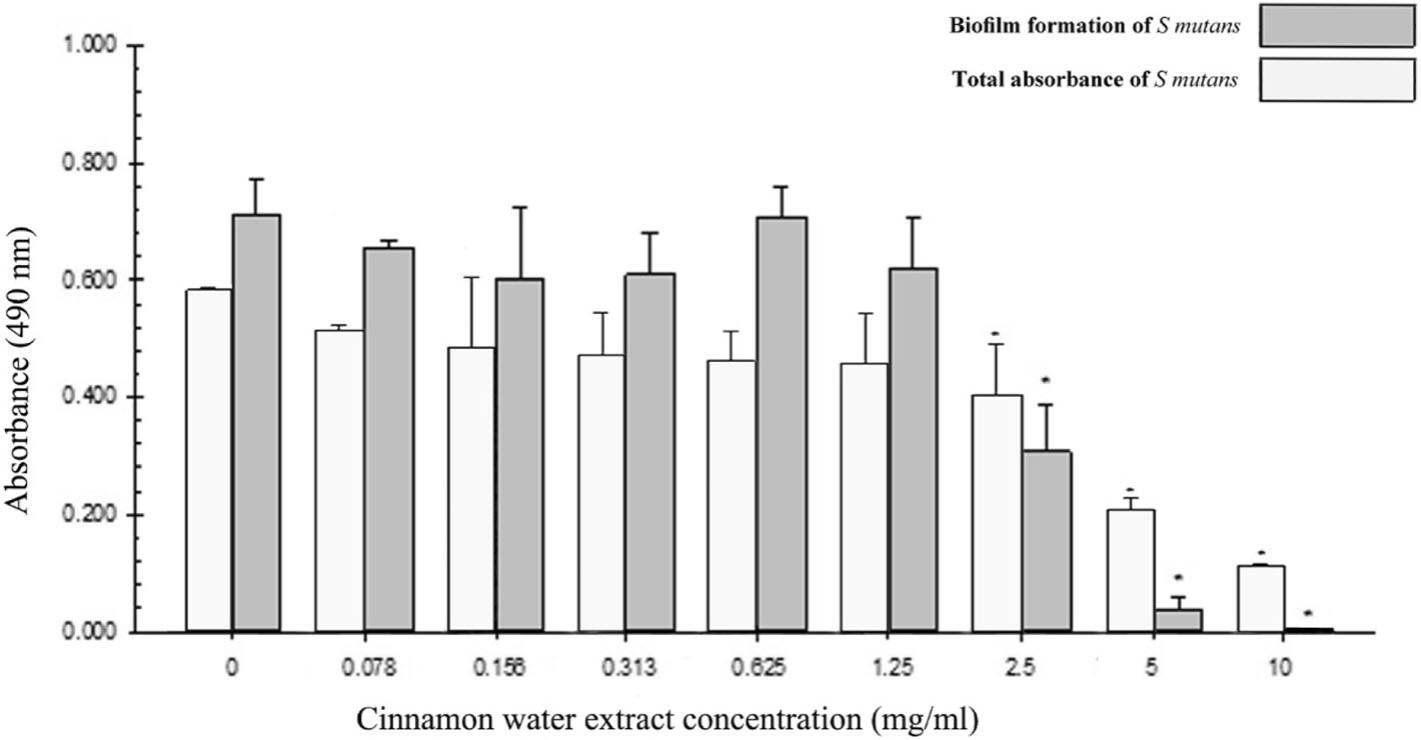
Effect of cinnamon water extract on total absorbance and biofilm formation of *S. mutans*. Asterisks indicate significant differences (*p* < 0.05) compared with samples without cinnamon

From the results of the preliminary experiment, the MIC of cinnamon water extract was determined to be 2.5 mg/ml (Fig. 1). The results also indicated that cinnamon was able to inhibit biofilm formation significantly (*p* < 0.05). There was significant reduction (*p* < 0.05) in biofilm formation between 2.5 to 10 mg/ml. It was seen clearly that 10 mg/ml was bactericidal, while 2.5 mg/ml cinnamon water extract exhibited significant biofilm formation inhibition (Fig. 1). For this reason, 2.5 mg/ml of cinnamon water extract was recognized as the MIC and the MBIC for *S. mutans* UA159.

### Results of the main experiment; effect of cinnamon water extract and nicotine

Overall, there was a significant effect of cinnamon water extract, nicotine, and their interaction in all measures examined (total absorbance, planktonic, biofilm).

**Fig. 2:**
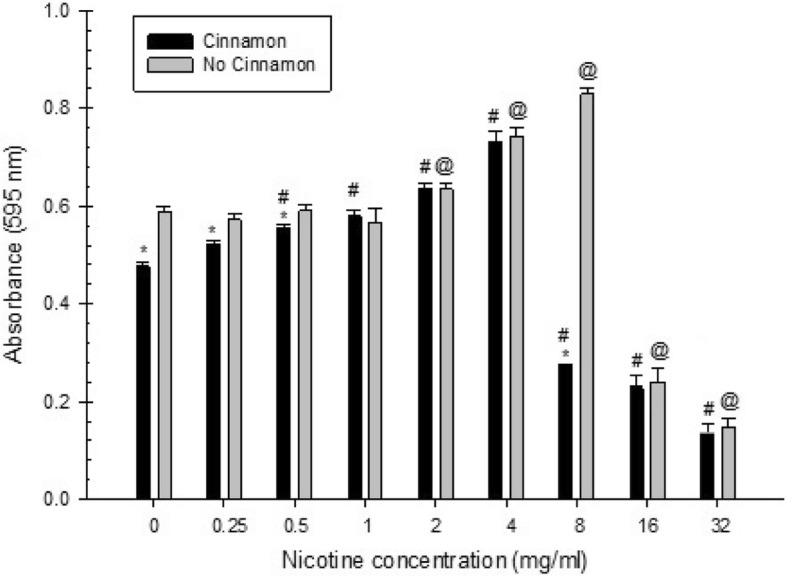
Combined effect of cinnamon water extract (2.5 mg/ml) and nicotine on *S. mutans* total absorbance. Asterisks indicate significant differences (*p* < 0.05) with cinnamon water extract compared to samples without cinnamon water extract. The # symbol indicates significant differences (*p* < 0.05) between *S. mutans* total absorbance with cinnamon water extract at different nicotine concentrations and the zero-nicotine concentration with cinnamon water extract. The @ symbol indicates significant differences (*p* < 0.05) between *S. mutans* total absorbance without cinnamon water extract at different nicotine concentrations and the zero-nicotine concentration without cinnamon water extract

**Fig. 3:**
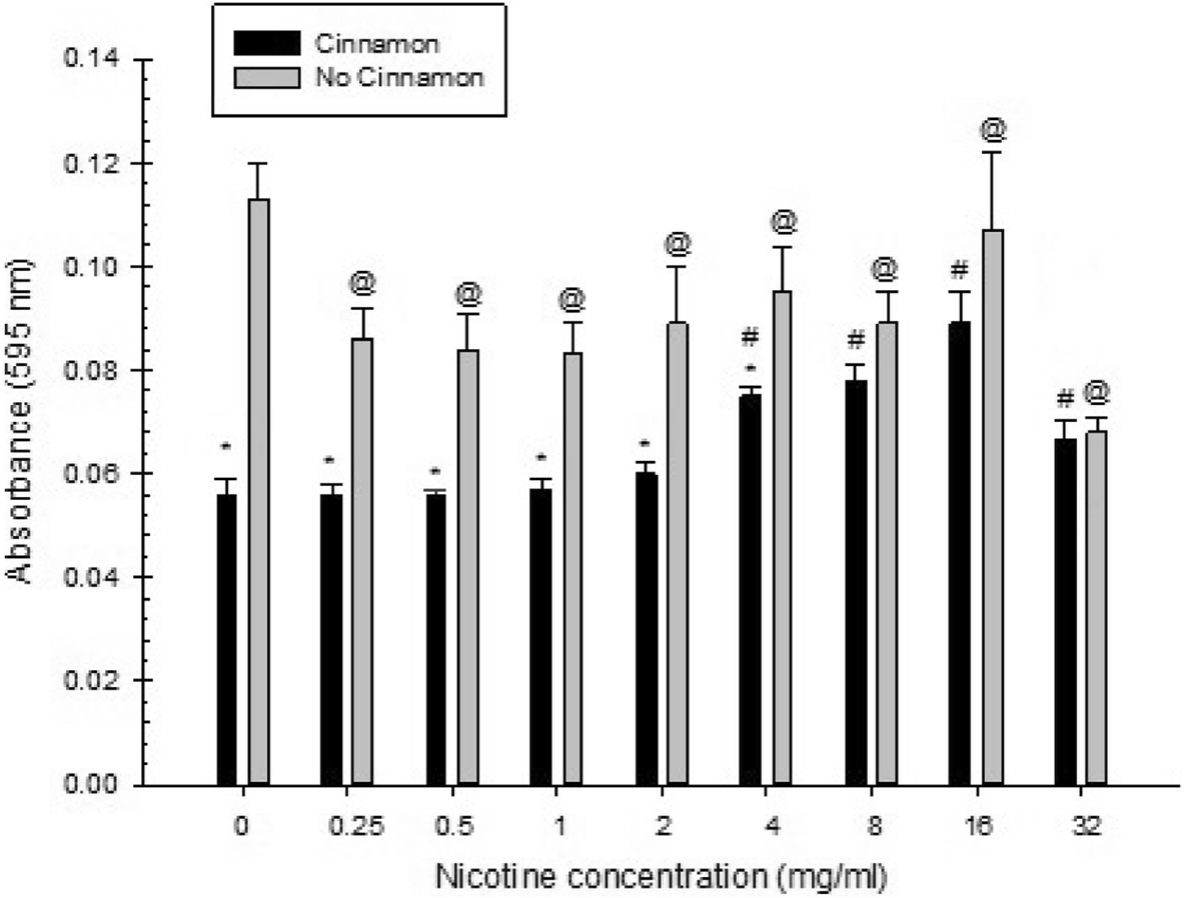
Combined effect of cinnamon (2.5 mg/ml) and nicotine on *S. mutans* planktonic growth. Asterisks indicate significant differences (*p* < 0.05) with cinnamon water extract compared to samples without cinnamon water extract. The # symbol indicates significant differences (*p* < 0.05) between *S. mutans* planktonic growth with cinnamon water extract at different nicotine concentrations and the zero-nicotine concentration with cinnamon water extract. The @ symbol indicates significant differences (*p* < 0.05) between *S. mutans* planktonic growth without cinnamon water extract at different nicotine concentrations and the zero-nicotine concentration without cinnamon water extract

**Fig. 4:**
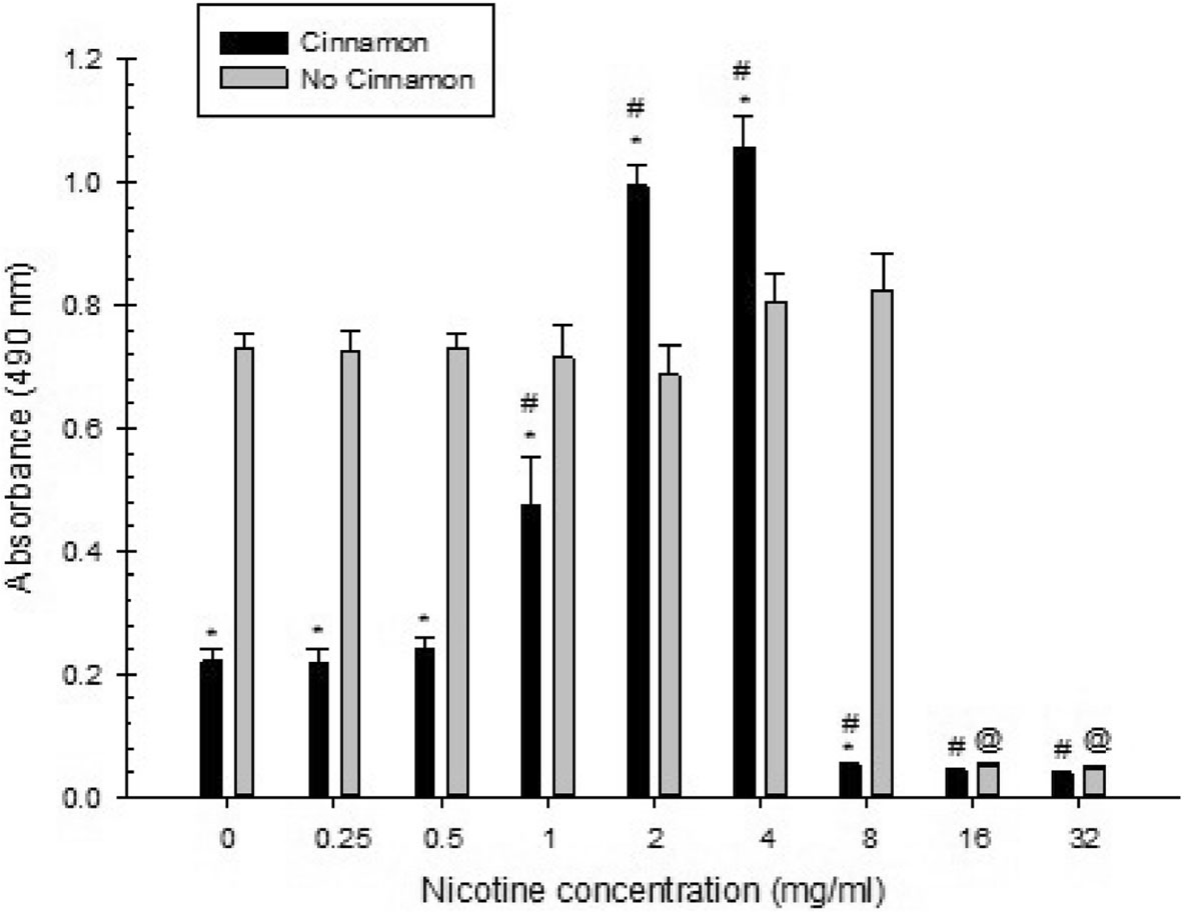
Combined effect of cinnamon water extract (2.5 mg/ml) and nicotine on *S. mutans* biofilm growth. Asterisks indicate significant differences (*p* < 0.05) with cinnamon water extract compared to samples without cinnamon water extract. The # symbol indicates significant differences (*p* < 0.05) between *S. mutans* biofilm growth with cinnamon water extract at different nicotine concentrations and the zero-nicotine concentration with cinnamon water extract. The @ symbol indicates significant differences (*p* < 0.05) between *S. mutans* biofilm growth without cinnamon water extract at different nicotine concentrations and the zero-nicotine concentration without cinnamon water extract

For total absorbance of *S. mutans* treated with nicotine and either with or without cinnamon water extract, there was a significant inhibitory effect of cinnamon water extract at 0, 0.25, 0.5 and 8 mg/ml of nicotine. However, a slight increase in total absorbance was observed at 1 and 2 mg/ml of nicotine, but these were not statistically significant. In addition, a non-statistically significant decrease in total absorbance was noted at 4, 16 and 32 mg/ml of nicotine (Fig. 2). Regarding planktonic growth, there was a significant inhibitory effect of cinnamon water extract at 0, 0.25, 0.5, 1, 2 and 4 mg/ml of nicotine. Also, at 8, 16 and 32 mg/ml of nicotine, there was a decrease in planktonic growth, but these were not statistically significant (Fig. 3). As for biofilm growth, there was a significant inhibitory effect of cinnamon water extract at 0, 0.25, 0.5, 1 and 8 mg/ml of nicotine, whereas a significant increase in biofilm growth was observed at 2 and 4 mg/ml of nicotine. At nicotine concentrations of 16 and 32 mg/ml, there was a decrease in biofilm growth, but these were not statistically significant (Fig. 4).

For total absorbance of *S. mutans* growth there was a significant increase with the presence of 2.5 mg/ml cinnamon water extract at 0.5, 1, 2 and 4 mg/ml of nicotine compared with the zero-nicotine concentration with cinnamon water extract. On the other hand, there was a significant decrease with the presence of cinnamon water extract at 8, 16 and 32 mg/ml of nicotine compared with the zero-nicotine concentration with cinnamon water extract. Also, there was a slight increase in total absorbance at 0.25 mg/ml of nicotine, but this was not statistically significant (Fig. 2). As for planktonic growth, there was a significant increase with the presence of cinnamon water extract at 4, 8, 16 and 32 mg/ml of nicotine compared with the zero-nicotine concentration with cinnamon water extract. However, a non-statistically significant slight increase was noted with the presence of cinnamon water extract at 2 mg/ml of nicotine compared with the zero-nicotine concentration with cinnamon water extract. In addition, there was no statistical significance with the presence of cinnamon water extract at nicotine concentrations of 0.25, 0.5 and 1 mg/ml compared with the zero nicotine concentration with cinnamon (Fig. 3). Regarding biofilm growth, there was a significant increase with the presence of cinnamon water extract at 1, 2 and 4 mg/ml of nicotine compared with the zero-nicotine concentration with cinnamon water extract, whereas, there was a significant decrease with the presence of cinnamon water extract at nicotine concentrations of 8, 16 and 32 mg/ml compared with the zero-nicotine concentration with cinnamon water extract. In addition, there was a slight increase in biofilm at 0.5 mg/ml of nicotine and a slight decrease at 0.25 mg/ml of nicotine, but this was not statistically significant (Fig. 4).

Regarding total absorbance of *S. mutans* growth there was a significant decrease without cinnamon water extract at 16 and 32 mg/ml of nicotine compared with the zero-nicotine concentration without cinnamon water extract. However, there was a significant increase without the presence of cinnamon water extract at 2, 4 and 8 mg/ml of nicotine compared with the zero-nicotine concentration without cinnamon water extract. Also, there was a decrease in biofilm at 0.25, 0.5 and 1 mg/ml of nicotine, but these were not statistically significant (Fig. 2). As for planktonic growth, there was a significant decrease without cinnamon water extract at all nicotine concentrations compared with the zero-nicotine concentration without cinnamon water extract (Fig. 3). For biofilm growth, there was a significant decrease without the presence of cinnamon water extract at 16 and 32 mg/ml of nicotine compared with the zero-nicotine concentration without cinnamon water extract. On the other hand, there was a slight increase in biofilm without cinnamon water extract at 4 and 8 mg/ml and a slight decrease at 1 and 2 mg/ml of nicotine. Both were not statistically significant compared with the zero-nicotine concentration without cinnamon water extract. In addition, there was no statistically significant difference at nicotine concentrations of 0.25 and 0.5 mg/ml (Fig. 4).

## Discussion

The preliminary experiment was designed for two reasons: 1) to confirm the antimicrobial activities of cinnamon water extract; and 2) to confirm the MIC of the specific cinnamon water extract used in this study. In this study, *C. burmannii* powder (also called Korintje, Padang cassia, Java, or Indonesian cinnamon) was used because it is the most common and least costly type of cinnamon sold in the US, including in grocery stores.

Polyphenols and volatile phenols are the main chemical compounds in cinnamon. With regard to polyphenols, cinnamon contains mainly caffeic, vanillic, gallic, p-coumaric, ferulic and cinnamic acids [21]. Among volatile components cinnamaldehyde is the most abundant substance in cinnamon and contribute to the fragrance and to the various biological activities observed with cinnamon [22]. Furthermore, the other minor volatile compounds are hydrocarbons and oxygenated compounds (e.g., β-caryophyllene, linalool, eugenyl acetate, and cinnamyl acetate) [23, 24].

Even though the antimicrobial activity of cinnamon was discussed in many previous studies, there is a lack of evidence regarding the direct effect of cinnamon on *S. mutans* biofilm growth. In addition, *S. mutans* is considered one of the normal flora species in the oral cavity. So, it is biologically relevant to obtain the MIC to not negatively affect the normal ecology of the oral flora. An overnight culture of *S. mutans* was placed in 96-well microtiter plates with TSBS to stimulate *S. mutans* growth and biofilm formation. Nicotine was added to both study and control groups to increase biofilm formation as this was confirmed in previous studies [5, 6, 20, 25].

The results of this study indicate that cinnamon water extract alone is able to significantly diminish the biofilm formation of *S. mutans* and also significantly limits the ability of nicotine to increase the growth of *S. mutans* at very low concentrations of nicotine (0.25 mg/ml, 0.5 mg/ml and 1 mg/ml) as well as also at very high levels of nicotine (8 mg/ml, 16 mg/ml and 32 mg/ml). However, the results indicate that cinnamon water extract is able to significantly enhance the ability of nicotine to increase the growth of *S. mutans* at certain nicotine concentrations (2 mg/ml and 4 mg/ml). This was interpreted to indicate that any nicotine concentration above 8 mg/ml, when combined with 2.5 mg/ml of cinnamon water extract can strongly inhibit *S. mutans* biofilm formation and any nicotine concentrations below 1 mg/ml also inhibit *S. mutans* biofilm formation. However, nicotine concentrations of 2 mg/ml and 4 mg/ml can enhance *S. mutans* biofilm formation.

The cinnamon water extract was investigated using microtiter plates. The plates contained the study groups of the cinnamon water extract/nicotine combinations and a control group of nicotine concentrations without cinnamon water extract, because it was better to include both study and control groups in the same microtiter plate to standardize the environmental and preparation conditions. A sterility group was added to assure that contamination was not present, because if there was contamination, the sterility wells will exhibit some kind of bacterial growth. A two-way ANOVA was used to compare the effects of the presence of 2.5 mg/ml cinnamon water extract and nicotine (concentrations ranging from 0 to 32 mg/ml) on *S. mutans* biofilm, planktonic cells, and total absorbance. Pairwise comparisons were made between nicotine concentrations to the zero-nicotine both with and without 2.5 mg/ml of cinnamon water extract. Pairwise comparisons were also made comparing each nicotine concentration with and without cinnamon water extract. Since the experimental trial was repeated 3 times (with 4 samples per group per repeat), a random effect for the multiple trials was used. Due to non-normality, a rank transformation was used prior to analysis.

There are different bacterial growth phases (planktonic and biofilm) among which the biofilm form is considered the most important phase, and the most favorable phase for oral bacteria to grow in vivo and cause disease. Moreover, protein expression in biofilm cells differs from the expression that is observed in planktonic cells [26]. So, even though cinnamon water extract demonstrated a minor restriction of nicotine activity when total absorbance and planktonic growth were measured, this restriction was recognized clearly when biofilm formation was assessed. In the nicotine group (without cinnamon), it was observed with increasing nicotine concentrations, there were increases in biofilm formation. Meanwhile with the 16 and 32 mg/ml nicotine concentrations, there were strong inhibitory effects on *S. mutans* growth and viability, respectively. These results confirm a previous study with seven strains of *S. mutans*, including the UA159 strain tested in this study, exposed to varying concentrations of nicotine [20]. It was theorized previously that any nicotine concentration above 8 mg/ml is toxic to *S. mutans*, and furthermore any concentration at or above 32 mg/ml is bactericidal.

It is understood that 2.5 mg/ml cinnamon water extract with 16 or 32 mg/ml of nicotine demonstrated more inhibition than nicotine alone and this is likely attributable to the combined antimicrobial effect of 2.5 mg/ml cinnamon water extract and nicotine. At 0.25, 0.5, 1 and 8 mg/ml nicotine concentrations, it was observed that without cinnamon water extract, there was more biofilm formation than for those with 2.5 mg/ml cinnamon water extract. However, a significant increase in bacterial growth was clearly seen when cinnamon water extract was added with concentrations of 2 mg/ml and 4 mg/ml nicotine. Two reasonable explanations are that nicotine at these concentrations overwhelmed the inhibitory effect of cinnamon water extract or that a synergistic effect developed between cinnamon water extract and nicotine at these specific concentrations of nicotine.

The clinical implication of these results can be related to generally incorporating cinnamon water extract with the appropriate concentration to oral hygiene products such as toothpaste, mouth washes and dental floss to inhibit *S. mutans* biofilm, and thereby inhibit or reduce caries incidence. Cinnamon is commonly added to oral hygiene products just as a flavoring agent and mouth refresher. However, based on the promising results of this study, in vivo studies are required to determine the dose to be used in products for oral hygiene, which have no cytotoxicity.

It is known that one cigarette contains nearly 1 mg of nicotine, which is partially absorbed into the bloodstream through the mucosal linings in the mouth with the remaining nicotine accumulating in saliva. According to one study, which measured the amount of nicotine in samples of saliva collected from smokers and non-smokers, the amount of nicotine in non-smokers’ saliva who are affected by secondary or tertiary hand smoke falls in the range of 0 to 0.31 mg/ml. In addition, it has been found that the amount of nicotine found in light or medium smokers’ saliva ranges from 0 to 1.33 mg/ml. Also, the amount of nicotine measured in heavy smokers’ saliva was in the range of 0 to 2.27 mg/ml [27]. Another study reported that the amount of nicotine in human saliva ranges from 0.07 to 1.56 mg/ml in saliva samples that were collected from smokers who have smoked for at least 10 years [28]. If we suppose that the average nicotine level in human saliva is 1 mg/ml, the cinnamon water extract demonstrated the ability to inhibit nicotine induced *S. mutans* biofilm at certain nicotine concentrations (0.25, 0.5, 1,8,16 and 32 mg/ml), so it could be said that cinnamon water extract added to oral hygiene products would be beneficial for smokers. In addition, some commercially available smoking cessation products such as chewing gum or lozenges contain cinnamon added as a flavoring agent and nicotine in amounts ranging from 2 mg to 4 mg of nicotine per lozenge and piece of chewing gum. The product is recommended to be used every two hours during the smoking cessation course and it is assumed that cinnamon water extract and nicotine accumulate in saliva and in turn is absorbed by biofilm. However, the last statement could be refuted because no prior study has investigated the amount of nicotine in the biofilm of smokers. Previous studies only reported the amount of nicotine in saliva. In-vivo studies are needed to determine the amount of nicotine in biofilm taken from smokers to assess the amount of nicotine absorbed by their biofilm. In addition, further research may be needed to investigate how cinnamon specifically inhibits *S. mutans* biofilm formation, and what effect cinnamon water extract may have on extracellular polysaccharide synthesis, glucosyltransferase synthesis, glucan-binding protein synthesis and acid production. Nicotine was found to increase extracellular polysaccharide (EPS) synthesis, GbpA expression, Gtfs expression, and lactic acid production in *S. mutans*. Cinnamon water extract may interfere with EPS synthesis, GbpA, Gtfs expression or lactic acid production. In conclusion, in-vivo studies are needed to confirm the anti-microbial effect of cinnamon and its biocompatibility with oral tissues. There are number of limitations to this study: no positive control was utilized in this study such as chlorohexidine. However, in other studies in our lab chlorhexidine at 0.12% totally inhibits *S. mutans* biofilm formation. In addition, cytotoxicity test should have been done to determine the dose to be used in products for oral hygiene which poses no cytotoxicity. Also, the value of IC50 should have been estimated. Only one strain of *S. mutans* was tested in this study. In other words, other strains of *S. mutans* may interact differently with the cinnamon water extract, however, this strain (UA159) has been fully sequenced and is routinely used by many laboratories throughout the world. Additionally, there were no saliva or salivary components utilized in this study. Moreover, only one species of cinnamon was tested in this study.

## Conclusion

This study indicates that cinnamon water extract at 2.5 mg/ml demonstrated strong anti-*S. mutans* properties by inhibiting *S. mutans* biofilm formation. Additionally, the growth of nicotine-induced *S. mutans* was diminished in the presence of cinnamon water extract. This promising in vitro data would require in vivo studies to determine the dose to be used in products for oral hygiene, which have no cytotoxicity, so both smokers and non-smokers may gain a dual benefit of a mouth-refreshing and a caries-preventing agent.

## Supplementary information

**Additional file 1.** Raw data used to determine the MIC and MBIC of cinnamon water extract on S. mutans biofilm and the Combined effect of cinnamon water extract (2.5 mg/ml) and nicotine on S. mutans total growth absorbance, planktonic growth and biofilm formation.

## Data Availability

The datasets used and/or analyzed during the current study are made available as supplementary files.

## Abbreviations

ACP: Amorphous calcium-phosphate
ANOVA: Analysis of variance
*C, burmannii*: *Cinnamomum burmannii*
*C, zeylanicum*: *Cinnamomum zeylanicum*
CFU: Colony-forming units
CO2: Carbon dioxide
CPP: Casein phosphopeptide
EPS: Extracellular polysaccharide synthesis
GbpA: Glucan binding protein
GTF: *Glucosyltransferases*
IC50: The half maximal inhibitory concentration
Ldh: Lactate Dehydrogenase
MBIC: Minimum biofilm inhibitory concentration
MIC: Minimum inhibitory concentration
NHANES: National Health and Nutrition Examination Survey
OD: Optical density
p-coumaric acid: *para*-*Coumaric* Acid
PSI: Pounds per square inch
PTS: Phosphotransferase system
*S, mutans*: *Streptococcus mutans*
TSB: Tryptic Soy broth
TSBS: Tryptic Soy broth supplemented with 1% sucrose
