# Supplementary material for: In vitro Cariostatic effects of cinnamon water extract on nicotine-induced Streptococcus mutans biofilm
Source: BMC Complement Med Ther. 2020 Feb 11;20:45. doi: 10.1186/s12906-020-2840-x (PMC7076877; doi:10.1186/s12906-020-2840-x)
Supplement: Supplementary file 1 — Additional file 1. Raw data used to determine the MIC and MBIC of cinnamon water extract on S. mutans biofilm and the Combined effect of cinnamon water extract (2.5 mg/ml) and nicotine on S. mutans total growth absorbance, planktonic growth and biofilm formation. [file 12906_2020_2840_MOESM1_ESM.pdf]

Raw data for preliminary experiment

- Before starting the main study, a preliminary experiment was conducted to determine the minimum inhibitory concentration (MIC) and the minimum biofilm inhibitory concentration (MBIC) of cinnamon water extract alone on the growth of *S. mutans* in tryptic soy broth supplemented with 1% sucrose (TSBS)
- Serial dilutions of cinnamon water extract in the supernatant (ranging in concentration from 0 to 10 mg/ml) were prepared in TSBS
- Ten µl of *S. mutans* in TSB was treated with 190 µl of the cinnamon water extract dilutions
- Each experiment was repeated three times

MIC Determination

**Control 0** cinnamon	**0.078** mg/ml	**0.156** mg/ml	**0.313** mg/ml	**0.625** mg/ml	**1.25** mg/ml	**2.5** mg/ml	**5** mg/ml	**10** mg/ml
0.890	0.750	0.710	0.703	0.699	0.690	0.297	0.120	0.060
0.870	0.720	0.730	0.699	0.612	0,670	0.220	0.114	0.050
0.820	0.757	0.728	0.650	0.660	0.680	0.330	0.119	0.070
0.790	0.663	0.780	0.702	0.610	0.695	0.370	0.090	0.040
0.820	0.640	0.693	0.670	0.730	0.640	0.288	0.094	0.044
0.860	0.590	0.610	0.580	0.690	0.670	0.303	0.130	0.015
0.795	0.670	0.619	0.530	0.670	0.612	0.390	0.121	0.030
0.730	0.635	0.730	0.670	0.663	0.660	0.388	0.080	0.020
0.812	0.659	0.630	0.650	0.602	0.590	0.320	0.122	0.015
0.805	0.648	0.580	0.690	0.714	0.699	0.230	0.185	0.060
0.860	0.690	0.566	0.709	0.625	0.560	0.295	0.140	0.067
0.814	0.715	0.620	0.680	0.612	0.670	0.319	0.095	0.023

MBIC Determination

Control 0 cinnamon	0.078 mg/ml	0.156 mg/ml	0.313 mg/ml	0.625 mg/ml	1.25 mg/ml	2.5 mg/ml	5 mg/ml	10 mg/ml
0.675	0.645	0.563	0.502	0.696	0.690	0.302	0.021	0.004
0.708	0.663	0.513	0.640	0.705	0.507	0.262	0.057	0.000
0.801	0.648	0.555	0.634	0.654	0.603	0.423	0.015	0.002
0.663	0.663	0.780	0.658	0.778	0.682	0.243	0.053	0.003
0.650	0.640	0.516	0.650	0.730	0.640	0.255	0.014	0.000
0.710	0.590	0.610	0.505	0.702	0.670	0.303	0.070	0.002
0.670	0.670	0.523	0.502	0.670	0.510	0.410	0.013	0.002
0.715	0.635	0.760	0.670	0.680	0.630	0.245	0.058	0.004
0.680	0.595	0.630	0.570	0.690	0.512	0.260	0.051	0.004
0.805	0.648	0.515	0.690	0.704	0.620	0.230	0.016	0.000
0.740	0.597	0.566	0.704	0.625	0.508	0.302	0.024	0.002
0.710	0.702	0.620	0.620	0.780	0.640	0.414	0.057	0.000

Raw data for the main study

-The MIC for cinnamon water extract was determined to be 2.5 mg/ml.

- A stock solution of cinnamon water extract at 2.5 mg/ml was prepared

- In order to measure the effect of cinnamon water extract on nicotine-treated *S. mutans*, serial dilutions of TSBS were prepared to yield 0, 0.25, 0.5, 1, 2, 4, 8, 16 and 32 mg/ml nicotine without cinnamon water extract, and 0, 0.25, 0.5, 1, 2, 4, 8, 16 and 32 mg/ml nicotine with the MIC dilution of cinnamon water extract - -
- One hundred ninety µl of TSBS at each nicotine concentration was aliquoted into wells of a sterile 96-well flat bottom microtiter plate

- Ten µl of a fresh overnight TSB culture of *S. mutans* was added to each well

- Each experiment was repeated three times

Total absorbance data

Group	Control 0 mg Nicotine	0.25 Nicotine	0.5 Nicotine	1 Nicotine	2 Nicotine	4 Nicotine	8 Nicotine	16 Nicotine	32 Nicotine
W/ Cinnamon	0.460	0.495	0.578	0.624	0.582	0.660	0.273	0.230	0.130
W/ Cinnamon	0.503	0.582	0.586	0.629	0.678	0.682	0.294	0.210	0.150
W/ Cinnamon	0.444	0.481	0.596	0.588	0.651	0.695	0.280	0.341	0.190
W/ Cinnamon	0.528	0.520	0.552	0.604	0.657	0.698	0.276	0.270	0.188

W/ Cinnamon	0.492	0.515	0.519	0.513	0.555	0.680	0.277	0.340	0.190
W/ Cinnamon	0.495	0.553	0.510	0.528	0.623	0.700	0.279	0.270	0.245
W/ Cinnamon	0.437	0.559	0.567	0.559	0.608	0.694	0.278	0.393	0.230
W/ Cinnamon	0.469	0.532	0.554	0.529	0.568	0.659	0.267	0.320	0.288
W/ Cinnamon	0.440	0.486	0.524	0.573	0.648	0.830	0.266	0.290	0.225
W/ Cinnamon	0.519	0.518	0.563	0.618	0.653	0.802	0.269	0.130	0.280
W/ Cinnamon	0.478	0.502	0.536	0.583	0.672	0.818	0.260	0.140	0.350
W/ Cinnamon	0.442	0.505	0.576	0.609	0.715	0.857	0.270	0.144	0.270
W/O Cinnamon	0.541	0.574	0.556	0.568	0.650	0.728	0.854	0.285	0.260
W/O Cinnamon	0.542	0.544	0.597	0.590	0.655	0.792	0.910	0.270	0.220
W/O Cinnamon	0.548	0.556	0.556	0.589	0.640	0.713	0.841	0.230	0.286
W/O Cinnamon	0.585	0.529	0.569	0.558	0.649	0.680	0.746	0.250	0.220
W/O Cinnamon	0.584	0.522	0.560	0.374	0.569	0.783	0.813	0.230	0.282
W/O Cinnamon	0.576	0.557	0.572	0.408	0.644	0.737	0.815	0.314	0.269
W/O Cinnamon	0.596	0.518	0.560	0.545	0.619	0.677	0.845	0.280	0.254
W/O Cinnamon	0.629	0.579	0.525	0.567	0.527	0.701	0.833	0.296	0.275

W/O Cinnamon	0.585	0.650	0.682	0.668	0.638	0.863	0.791	0.260	0.273
W/O Cinnamon	0.625	0.604	0.668	0.653	0.711	0.757	0.827	0.240	0.245
W/O Cinnamon	0.611	0.609	0.600	0.651	0.691	0.755	0.838	0.285	0.230
W/O Cinnamon	0.643	0.621	0.636	0.640	0.609	0.768	0.839	0.275	0.195

Planktonic growth data

Group	Control 0 mg Nicotine	0.25 Nicotine	0.5 Nicotine	1 Nicotine	2 Nicotine	4 Nicotine	8 Nicotine	16 Nicotine	32 Nicotine
W/ Cinnamon	0.077	0.063	0.063	0.061	0.067	0.091	0.089	0.099	0.067
W/ Cinnamon	0.054	0.058	0.063	0.062	0.066	0.078	0.087	0.109	0.067
W/ Cinnamon	0.054	0.055	0.056	0.057	0.065	0.075	0.081	0.089	0.067
W/ Cinnamon	0.055	0.058	0.059	0.061	0.071	0.075	0.083	0.093	0.080
W/ Cinnamon	0.053	0.054	0.049	0.055	0.062	0.080	0.095	0.111	0.069
W/ Cinnamon	0.053	0.060	0.055	0.058	0.066	0.079	0.081	0.106	0.068
W/ Cinnamon	0.065	0.066	0.056	0.051	0.063	0.074	0.073	0.107	0.080

W/ Cinnamon	0.062	0.055	0.057	0.055	0.060	0.073	0.070	0.110	0.070
W/ Cinnamon	0.044	0.048	0.050	0.071	0.053	0.071	0.060	0.074	0.066
W/ Cinnamon	0.056	0.051	0.054	0.053	0.051	0.069	0.089	0.070	0.060
W/ Cinnamon	0.048	0.048	0.049	0.051	0.041	0.054	0.071	0.056	0.082
W/ Cinnamon	0.050	0.055	0.052	0.051	0.057	0.076	0.061	0.049	0.070
W/O Cinnamon	0.102	0.072	0.076	0.077	0.077	0.085	0.087	0.223	0.088
W/O Cinnamon	0.105	0.110	0.076	0.078	0.079	0.093	0.097	0.127	0.070
W/O Cinnamon	0.102	0.075	0.090	0.081	0.079	0.085	0.096	0.121	0.070
W/O Cinnamon	0.110	0.108	0.109	0.114	0.209	0.189	0.132	0.113	0.087
W/O Cinnamon	0.089	0.064	0.074	0.060	0.080	0.100	0.077	0.108	0.065
W/O Cinnamon	0.114	0.079	0.062	0.065	0.086	0.100	0.094	0.099	0.100
W/O Cinnamon	0.164	0.081	0.058	0.077	0.087	0.086	0.094	0.095	0.103
W/O Cinnamon	0.159	0.114	0.112	0.114	0.097	0.111	0.106	0.076	0.062
W/O Cinnamon	0.089	0.058	0.083	0.066	0.063	0.070	0.052	0.048	0.075
W/O Cinnamon	0.088	0.079	0.073	0.072	0.068	0.083	0.068	0.168	0.083
W/O Cinnamon	0.119	0.079	0.061	0.076	0.081	0.061	0.061	0.045	0.054

W/O Cinnamon	0.119	0.108	0.139	0.115	0.067	0.076	0.100	0.059	0.093

Biofilm formation data

Group	Control 0 mg Nicotine	0.25 Nicotine	0.5 Nicotine	1 Nicotine	2 Nicotine	4 Nicotine	8 Nicotine	16 Nicotine	32 Nicotine
W/ Cinnamon	0.211	0.284	0.209	0.387	0.699	1.011	0.057	0.048	0.053
W/ Cinnamon	0.339	0.263	0.172	0.897	1.095	1.027	0.054	0.055	0.046
W/ Cinnamon	0.164	0.114	0.264	0.337	1.058	0.804	0.051	0.049	0.047
W/ Cinnamon	0.141	0.139	0.225	0.528	1.068	0.950	0.055	0.054	0.056
W/ Cinnamon	0.211	0.283	0.318	0.472	1.008	1.162	0.060	0.066	0.045
W/ Cinnamon	0.207	0.289	0.183	0.593	1.115	1.153	0.058	0.054	0.046
W/Cinnamon	0.399	0.297	0.288	0.861	1.034	1.116	0.062	0.048	0.045
W/ Cinnamon	0.166	0.299	0.320	0.839	1.086	1.222	0.059	0.055	0.046
W/ Cinnamon	0.212	0.132	0.276	0.160	0.915	0.879	0.048	0.045	0.045
W/ Cinnamon	0.212	0.179	0.216	0.215	0.970	0.907	0.045	0.048	0.047
W/ Cinnamon	0.195	0.177	0.199	0.215	0.934	1.012	0.043	0.051	0.045
W/ Cinnamon	0.195	0.180	0.243	0.193	0.957	1.458	0.046	0.050	0.050

W/O Cinnamon	0.709	0.862	0.854	0.772	0.773	0.773	0.577	0.051	0.051
W/O Cinnamon	0.645	0.659	0.752	0.796	0.615	0.988	1.295	0.054	0.051
W/O Cinnamon	0.695	0.638	0.724	0.799	0.699	0.654	0.579	0.061	0.058
W/O Cinnamon	0.632	0.640	0.639	0.668	0.732	0.676	0.859	0.049	0.054
W/O Cinnamon	0.820	0.866	0.868	0.961	0.775	1.043	0.948	0.059	0.066
W/O Cinnamon	0.803	0.850	0.784	0.961	0.978	1.036	0.767	0.067	0.064
W/O Cinnamon	0.798	0.849	0.671	0.877	0.834	0.893	0.713	0.075	0.081
W/O Cinnamon	0.854	0.721	0.709	0.772	0.826	0.900	0.665	0.066	0.095
W/O Cinnamon	0.652	0.661	0.740	0.510	0.499	0.704	1.060	0.052	0.054
W/O Cinnamon	0.590	0.698	0.684	0.509	0.509	0.651	0.793	0.046	0.049
W/O Cinnamon	0.865	0.556	0.760	0.530	0.512	0.671	0.986	0.044	0.052
W/O Cinnamon	0.676	0.695	0.588	0.415	0.498	0.686	0.651	0.047	0.052
